# Extension of *Drosophila* lifespan by Korean red ginseng through a mechanism dependent on dSir2 and insulin/IGF-1 signaling

**DOI:** 10.18632/aging.102387

**Published:** 2019-10-31

**Authors:** Shin-Hae Lee, Hye-Yeon Lee, Mira Yu, Eunbyul Yeom, Ji-Hyeon Lee, Ah Yoon, Kyu-Sun Lee, Kyung-Jin Min

**Affiliations:** 1 Department of Biological Sciences, Inha University, Incheon 22212, Korea; 2 Metabolism and Neurophysiology Research Group, KRIBB, Daejeon 34141, Korea; 3 Department of Functional Genomics, UST, Daejeon 34141, Korea

**Keywords:** korean red ginseng, lifespan, *Drosophila melanogaster*, Sir2, insulin/IGF-1 signaling

## Abstract

Many studies have indicated that Korean red ginseng (KRG) has anti-inflammatory and anti-oxidative effects, thereby inducing many health benefits in humans. Studies into the longevity effects of KRG are limited and have provided contradictory results, and the molecular mechanism of lifespan extension by KRG is not elucidated yet. Herein, the longevity effect of KRG was investigated in *Drosophila melanogaster* by feeding KRG extracts, and the molecular mechanism of lifespan extension was elucidated by using longevity-related mutant flies. KRG extended the lifespan of *Drosophila* when administrated at 10 and 25 μg/mL, and the longevity benefit of KRG was not due to reduced feeding, reproduction, and/or climbing ability in fruit flies, indicating that the longevity benefit of KRG is a direct effect of KRG, not of a secondary artifact. Diet supplementation with KRG increased the lifespan of flies on a full-fed diet but not of those on a restricted diet, and the longevity effect of KRG was diminished by the mutation of *dSir2*, a deacetylase known to mediate the benefits of dietary restriction. Similarly, the longevity effect of KRG was mediated by the reduction of insulin/IGF-1 signaling. In conclusion, KRG extends the lifespan of *Drosophila* through Sir2 and insulin/IGF-1 signaling and has potential as an anti-aging dietary-restriction mimetic and prolongevity supplement.

## INTRODUCTION

Due to the various limitations of synthetic drugs, interest in complementary and alternative medicines (CAMs) is increasing worldwide, and ginseng is a popular CAM [1]. Ginseng root has been used for over 2,000 years as a traditional medicine in East Asia, and its efficacy was known in Western countries in the 18^th^ century [2]. The major species of commercially utilized ginseng are *Panax ginseng* (Asian ginseng), *Panax quinquefolius* (American ginseng), *Panax notoginseng* (Chinese ginseng), and *Panax japonicas* (Japanese ginseng). Among them, *P. ginseng* is the most actively studied for its beneficial health effects [3]. The root of *P. ginseng* has been reported to have clinical efficacy in several aging-related diseases such as cancer, diabetes, hyperlipidemia, hypertension, and dementia [4–6]. In addition, the leaves and fruits of ginseng have recently been reported to have anti-cancer and anti-senescence effects [7, 8]. The pharmaceutical bioactive component of ginseng has been shown to be saponin, a so-called ginsenoside, but recently, non-saponin components of ginseng such as polysaccharides have also been reported to have nutraceutical effects [9].

Red ginseng is produced by steaming followed by drying of ginseng root. Heat treatment, at a reasonable temperature, on fresh ginseng boosts its pharmaceutical effects mainly due to the transformation and degradation of ginsenosides [10–12]. The steaming process decreases the concentrations of polar protopanaxadiol ginsenosides (such as Rg1, Rb1, Rb2, Re, Rc, and Rd) and increases the concentrations of less polar ginsenosides (such as Rg3, Rg5, Rh1, and Rh2) [13, 14]. In particular, ginsenosides Rg3 and Rg5, reported to have strong anti-cancer activity, are detected in red ginseng but not in fresh ginseng [13, 15]. Steaming also changes the structures of other constituents of ginseng like flavonoids, polyacetylenes, phytosterols, essential oils, polysaccharides, and vitamins [13]. In addition, amino sugars such as arginine-fructose-glucose and arginine-fructose, and acidic polysaccharides are increased by the red ginseng production process.

Traditional remedy claims in oriental medicine include a longevity-promoting effect of ginseng, and several studies have investigated the effect of ginseng on lifespan in several model organisms. Yu et al. [16] showed that supplementation of *P. ginseng* extended the mean lifespan of *Caenorhabditis elegans* by 7.7%. In addition, Lee et al. [17] reported that supplementation with 300 μg/mL of total saponins from *P. ginseng* extended the maximum lifespan of *C. elegans*, and ginsenoside Rc was the main component of its prolongevity effect. Likewise, supplementation with polysaccharides from *P. notoginseng* at 0.1 mg/mL extended the lifespan of *C. elegans* by 5% - 21% [18]. In contrast to the consistent prolongevity effect of ginseng in *C. elegans*, the effect in other model organisms and humans is unclear. Massie and Williams [19] showed that supplementation with *P. ginseng* at 0.25 mg/mL did not change the lifespan of Oregon-R *Drosophila*, and supplementation at 2.5 or 25 mg/mL rather decreased the survival of fruit flies. Similarly, Bittles et al. [20] showed that oral administration of *P. ginseng* at 40 mg/kg/day to 8- or 52-week-old mice did not alter their lifespan. Finally, an 18 year, population-based cohort study in Kangwha county in Korea showed that ginseng intake decreased all-cause mortality in older men, but not in women [21].

Recently, Kim [22] tested the longevity effect of red ginseng and reported that 1.2 and 12 μg/mL of Korean red ginseng (KRG) tonic extended the mean lifespan of the *w^1118^ Drosophila* strain by 14.5% and 13.5%, respectively. However, the KRG tonic used in that study (Hongsamton Mild^®^) contains other nutraceutical ingredients including Korean angelica root, mistletoe, rehmania, and hartshorn extract [22]; thus, the longevity effect of the KRG tonic may not have been solely caused by KRG. For example, mistletoe extract, one of the components in KRG tonic, increases the lifespan of *Drosophila* [23].

As mentioned, there is no definitive evidence of a prolongevity effect of ginseng. Moreover, the molecular mechanisms related to a possible lifespan extension by ginseng have not been elucidated. In this study, we investigated the longevity effect and underlying mechanism of lifespan extension of KRG in *Drosophila melanogaster*. The results indicate that KRG extends the lifespan of *D.* *melanogaster* through Sir2 and insulin/IGF-1 signaling (IIS). Our results suggest that KRG might be an excellent candidate as an anti-aging supplement and geroprotector [24].

## RESULTS

### Lifespan-extending effect of KRG

**Figure 1:**
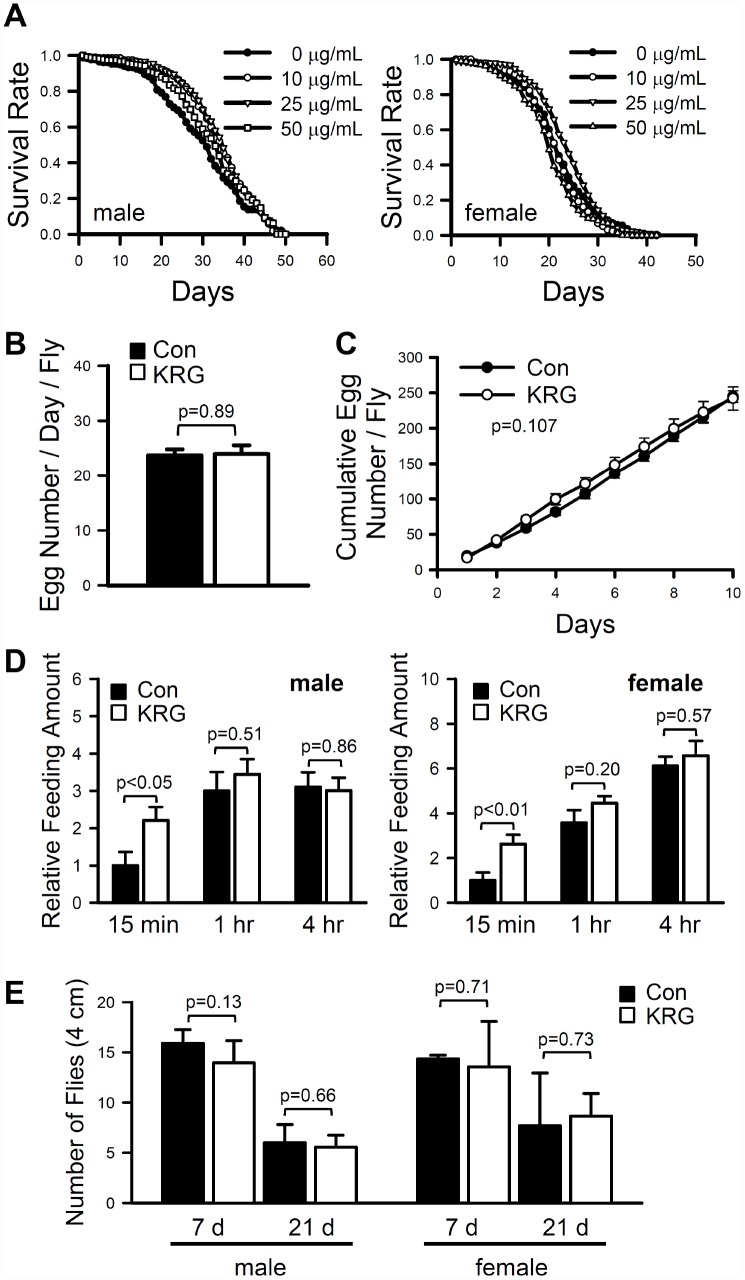
**Effect of KRG on lifespan in fruit flies.** (**A**) Survival of flies fed a KRG-containing diet (lines with open dots) or a control diet (lines with closed dots). Fecundity (**B**, **C**), amount of food intake (**D**), and locomotive activity (**E**) of fruit flies fed a KRG-containing diet or a control diet.

To investigate the effect of KRG on the fruit fly lifespan, the lifespan of wild-type strain CS_10_ flies was measured after feeding KRG at concentrations ranging from 10 to 50 μg/mL. The mean lifespan of flies fed a KRG-supplemented diet was significantly increased compared to that of control flies (Figure 1A). In males, the mean lifespan of flies fed a 10 or 25 μg/mL KRG supplement was 33.86 ± 0.57 days (a 13.28% increase, log-rank test χ^2^ = 4.64, *p* < 0.05) and 34.18 ± 0.56 days (a 14.35% increase, log-rank test, χ^2^ = 8.16, *p* < 0.005), respectively, while that of control flies was 29.89 ± 0.70 days (Supplementary Table 1). In female flies, supplementation with 25 μg/mL KRG increased the mean lifespan from 22.01 ± 0.42 days to 23.81 ± 0.40 days (an 8.18% increase, log-rank test χ^2^ = 5.63, *p* < 0.05) (Figure 1A and Supplementary Table 1). Notably, the high concentration of KRG (50 μg/mL) decreased the survival rate of female flies (Figure 1A and Supplementary Table 1, an 11.27% decrease, log-rank test χ^2^ = 24.60, *p* < 0.0001). These results indicate that a dietary KRG supplement at an optimal concentration range of 10-25 μg/mL extends the lifespan of fruit flies; thus, the 25 μg/mL KRG treatment was used in subsequent experiments.

Organismal lifespan can be increased by changes in physiological status, such as decreased reproductive output or reduced food intake, and can be accompanied with reduced climbing ability in flies [25, 26]. To verify whether the observed lifespan extension was truly due to the KRG’s lifespan-extending effect or was caused by changes in physiological status, the fecundity and feeding rate of flies fed a KRG-supplemented diet were measured. The number of eggs laid by control flies per day was 23.7 ± 1.13, while that of KRG supplement-fed flies was 24 ± 1.61 (Figure 1B, *t*-test DF = 38, *p* = 0.89; Figure 1C, two-way repeated measures ANOVA F = 2.734, *p* = 0.107). In addition, the amount of food consumed by KRG-pretreated flies was not decreased compared to that consumed by control flies, regardless of sex (Figure 1D, male, *t*-test, 15 min DF = 18, *p* < 0.05, 1 h DF = 18, *p* = 0.51, 4 h DF = 18, *p* = 0.86; female, *t*-test, 15 min DF = 18, *p* < 0.01, 1 h DF = 18, *p* = 0.20, 4 h DF = 18, *p* = 0.57). Likewise, KRG supplementation did not alter the climbing ability of flies of either sex (Figure 1E, male, *t*-test, 7 days, DF = 8, *p* = 0.13; 21 days, DF = 4, *p* = 0.66; female, 7 days, DF = 8, *p* = 0.71; 21 days, DF = 5, *p* = 0.73). These results suggest that KRG has potential as a lifespan-extending dietary supplement with no adverse effects on physical activity.

### Effects of KRG on stress resistance

**Figure 2:**
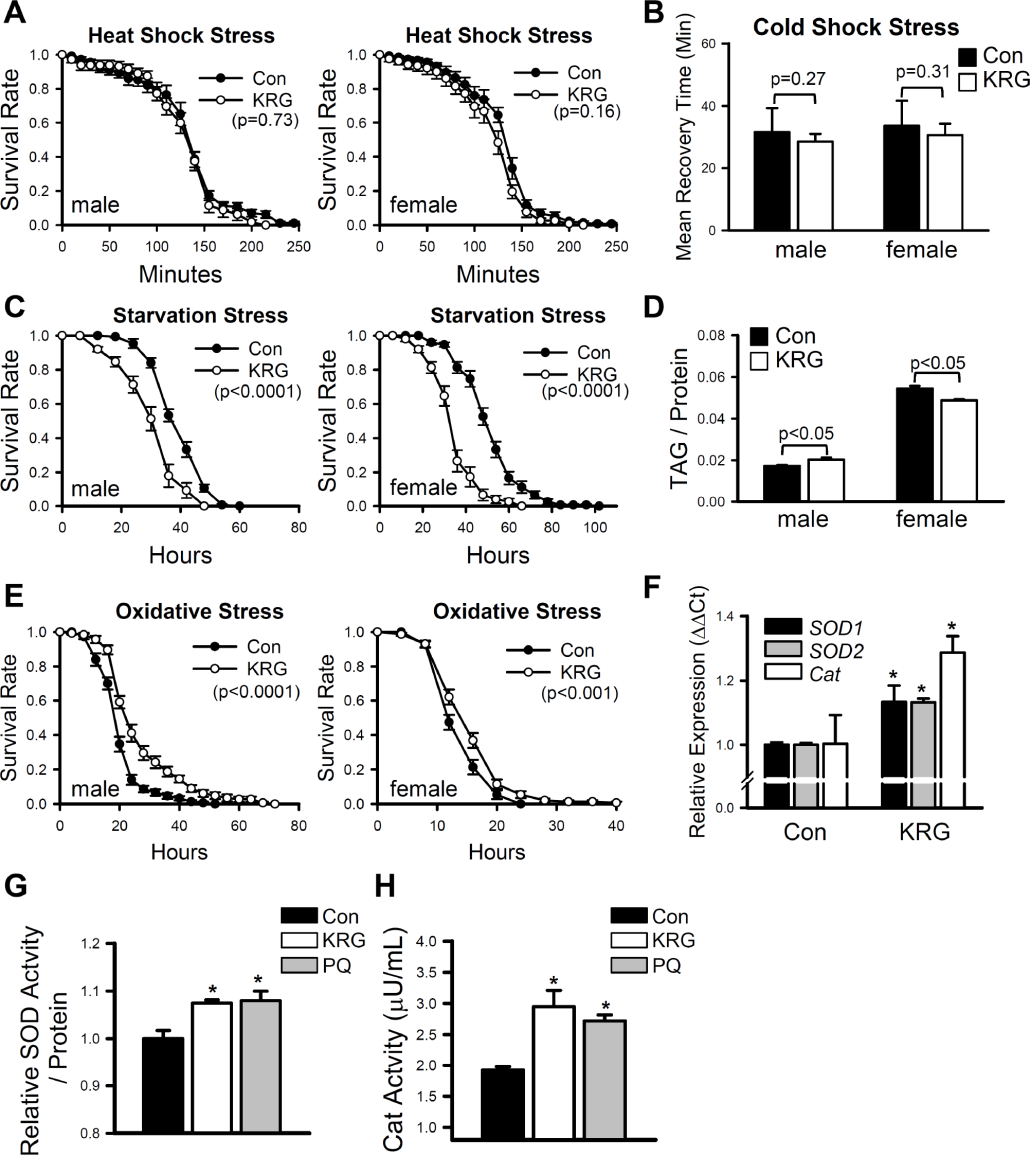
**Effect of KRG on stress resistance in fruit flies.** Survival of fruit flies fed a KRG-containing diet or a control diet under heat-shock stress (**A**), starvation stress (**C**), and oxidative stress (**E**). (**B**) Recovery time of flies fed a KRG-containing diet or a control diet after cold-shock (freezing). (**D**) TAG level of flies fed a KRG-containing diet or a control diet. (**F**) The mRNA levels of antioxidant enzymes were analyzed in fruit flies fed a KRG-containing diet or a control diet for 7 or 40 days. Enzyme activity of SOD (**G**) or Cat (**H**) was measured after KRG or positive control paraquat treatment. *p < 0.05.

Many pharmaceutical and nutraceutical products that have life-extending effects can also increase resistance to various environmental stresses [27, 28]. To verify the effect of KRG on stress resistance, flies pretreated with KRG for 7 days were exposed to thermal, starvation, and oxidative stresses. Resistance to thermal stresses (heat-shock and cold-shock) was not affected by the KRG supplementation (Figure 2A, heat-shock, male, log-rank test χ^2^ = 0.16, *p* = 0.73; female, log-rank test χ^*2*^ = 2.02, *p* = 0.16; Figure 2B, cold-shock, *t*-test, male DF = 11, *p* = 0.27; female, DF = 13, *p* = 0.31). In contrast, resistance to starvation stress was markedly decreased by KRG supplementation in both males and females (Figure 2C, male, 22% decrease, log-rank test χ^2^ = 58.61, *p* < 0.0001; female, 32% decrease, log-rank test χ^2^ = 111.18, *p* < 0.0001). Since the survival rate under starvation conditions is largely affected by the amount of stored fat [29], we measured the amount of TAG, the primary lipid-storage molecule in insects [29]. KRG supplementation decreased the TAG level of young females (Figure 2D, 8.02% decrease, *t*-test DF = 4, *p* < 0.05); however, a decrease in TAG level was not observed in males (Figure 2D, 10.7% increase, *t*-test DF = 4, *p* < 0.05). The dehydration level was examined since it can affect metabolic response. Similar to the TAG results, the water content was unchanged by KRG supplementation in male flies (Supplementary Figure 1, *t*-test, DF =28, *p* = 0.11), but was mildly decreased in females (Supplementary Figure 1, 1.9% decrease, *t*-test DF = 28, *p* < 0.01). Under the paraquat-induced oxidative stress condition, KRG supplementation increased the survival of both male and female flies (Figure 2E, male, 33.1% increase, log-rank test χ^2^ = 34.52, *p* < 0.0001; female, 12.5% increase, log-rank test χ^2^ = 10.93, *p* < 0.001), suggesting that KRG can confer oxidation resistance, a result that is consistent with those in previous reports [6, 30]. To verify the antioxidant activity of KRG, the expression levels of antioxidant enzymes in flies fed a KRG supplement were measured. The expression levels of the antioxidant enzymes *Cu/Zn-SOD* (*SOD1*), *Mn-SOD* (*SOD2*), and *catalase* (*Cat*) were increased by KRG supplementation (Figure 2F). Likewise, the enzymatic activity levels of SOD and Cat were elevated after KRG treatment (Figure 2G and 2H).

### Lifespan-extending effect of KRG and dietary restriction

**Figure 3:**
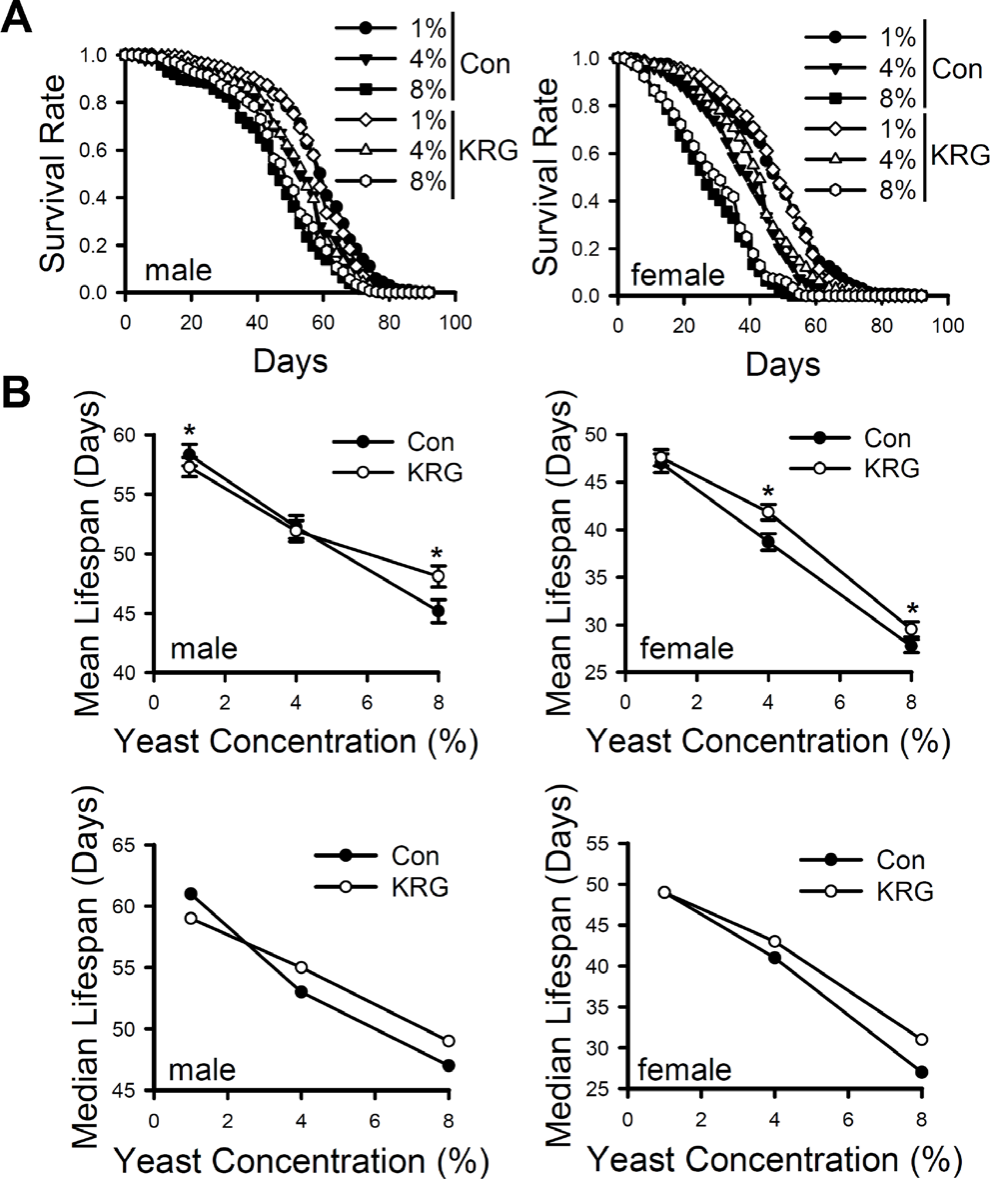
**Effect of KRG on the lifespan of fruit flies fed a DR diet.** (**A**) The survival of flies fed KRG-supplemented 1%, 4%, or 8% yeast extract diets (lines with open dots) or control diets (lines with closed dots). (**B**) The mean (upper graphs) and median (lower graphs) lifespans are shown. *p < 0.05.

Dietary restriction (DR) is a well-established strategy to extend an organism's lifespan [31], and several nutraceutical products are reported to extend the lifespan of animals via a mechanism similar to that in DR; that is, the products act as a DR mimetic [31]. To determine whether KRG functions as a DR mimetic, fruit flies were fed 1%, 4%, or 8% yeast extract diet with/without the addition of KRG. Male fruit flies fed 1% or 4% yeast diet had 29.03% and 15.62% higher survival than that of male flies fed the 8% yeast diet (Figure 3A left panel and Supplementary Table 2, 1% *vs.* 8% diet, log-rank test χ^2^ = 22.16, *p* < 0.0001; 4% *vs.* 8% diet, log-rank test χ^2^ = 104.35, *p* < 0.0001). DR via the yeast-supplemented diet also increase survival in females (Figure 3A right panel and Supplementary Table 2, 1% *vs.* 8% diet, 69.21% increase, log-rank test χ^2^ = 51.41, *p* < 0.0001; 4% *vs.* 8% diet, 37.6% increase, log-rank test χ^2^ = 252.06, *p* < 0.0001). KRG supplementation significantly increased the mean lifespan of males flies fed the 8% yeast diet (Figure 3B left panel and Supplementary Table 2, 6.46% increase, log-rank test χ^2^ = 4.58, *p* < 0.05), as well as that of female flies fed the 4% or 8% yeast diet (Figure 3B right panel and Supplementary Table 2, 4% diet, 9.55% increase, log-rank test χ^2^ = 6.16, *p* < 0.05; 8% diet, 6.41% increase, log-rank test χ^2^ = 6.28, *p* < 0.05). However, KRG supplementation failed to increase the mean lifespan of flies fed the 1% yeast diet; lifespan significantly decreased in males, but no change was detected in females (Figure 3B upper panel and Supplementary Table 2; males, 1.75% decrease, log-rank test χ^2^ = 6.34, *p* < 0.05; females, log-rank test χ^2^ = 0.53, *p* = 0.47). In addition, median lifespan was increased by KRG supplementation but only in flies fed the 4% and 8% diets (Figure 3B lower panel and Supplementary Table 2). These results indicate that KRG can extend the lifespan of fruit flies in a manner similar to that of DR.

### dSir2 dependency of KRG-induced life extension

**Figure 4:**
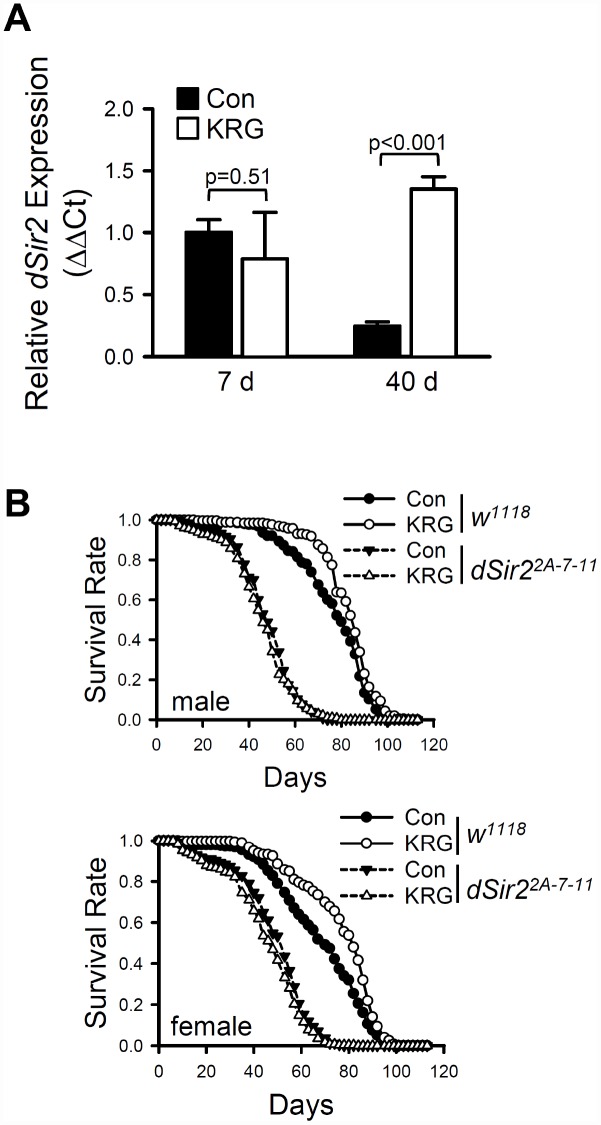
**Lifespan extension by KRG is mediated through the Sir2 pathway.** (**A**) The mRNA level of *dSir2* was analyzed in the whole body of male fruit flies fed a KRG-containing diet or a control diet for 7 or 40 days. (**B**) Survival of *dSir2* null mutant flies (*dSir2^2A-7-11^*, dashed lines) and control flies (*w^1118^*, solid lines) fed a KRG-containing diet (lines with open dots) or a control diet (lines with closed dots).

Although the mechanisms underlying lifespan extension by DR have not yet been fully elucidated, it has been suggested that lifespan extension by DR is mediated by activation of Sirtuin 1 (Sirt1), a NAD+ dependent deacetylase [32]. To determine whether the KRG-induced life-extending effect is associated with dSir2, the *Drosophila* homolog of Sirt1, the expression level of *dSir2* was measured after flies ingested KRG for 7 or 40 days. The mRNA level of *dSir2* was not significantly changed after KRG supplementation for 7 days (Figure 4A, *t*-test DF = 2, *p* = 0.51); however, the aging-related decline of *dSir2* expression was significantly suppressed by ingestion of KRG for 40 days. Approximately 5.4 times higher levels of *dSir2* mRNA were detected in the 40-day-old KRG-fed flies than in the 40-day-old control flies (Figure 4A, *t*-test DF = 3, *p* < 0.001).

To examine directly the requirement for dSir2 activation in the life-extending effect of KRG, survivals of *dSir2* null mutant (*dSir2^2A-7-11^*) and its genetic background control (*w^1118^*) flies fed a KRG supplement were determined. Consistent with the results for flies of the CS_10_ strain, KRG supplementation increased the lifespan of the long-lived *w^1118^* flies (Figure 4B and Supplementary Table 3, male, 8.68% increase, log-rank test χ^2^ = 18.42, *p* < 0.0001; female, 13.21% increase, log-rank test χ^2^ = 28.89, *p* < 0.0001), and decreased the hazard ratio (Cox proportional hazard analysis, male, HR = 0.665, *p* < 0.0001; female, HR = 0.709, *p* < 0.0001). However, KRG supplementation failed to increase the lifespan of *dSir2^2A-7-11^* flies; rather, while KRG supplementation did not change the lifespan of male *dSir2^2A-7-11^* flies (Figure 4B and Supplementary Table 3, log-rank test χ^2^ = 1.17, *p* = 0.28), it did decrease the lifespan of female *dSir2^2A-7-11^* flies (Figure 4B and Supplementary Table 3, 6.28% decrease, log-rank test χ^2^ = 6.39, *p* < 0.05). The increased hazard ratio shown by *dSir2* mutation was decreased by KRG supplementation (Cox proportional hazard analysis, *dSir2* mutation, male HR = 4.152, *p* < 0.0001, female HR = 10.642, *p* < 0.0001; KRG+*dSir2* mutation, male HR = 1.848, *p* < 0.0001, female HR = 1.515, *p* < 0.0001). Taken together, it is concluded that the lifespan-extending effect by KRG is mediated by dSir2 in a manner similar to that of DR.

### Relationship between KRG-induced life extension and the insulin/IGF-1 signaling pathway

**Figure 5:**
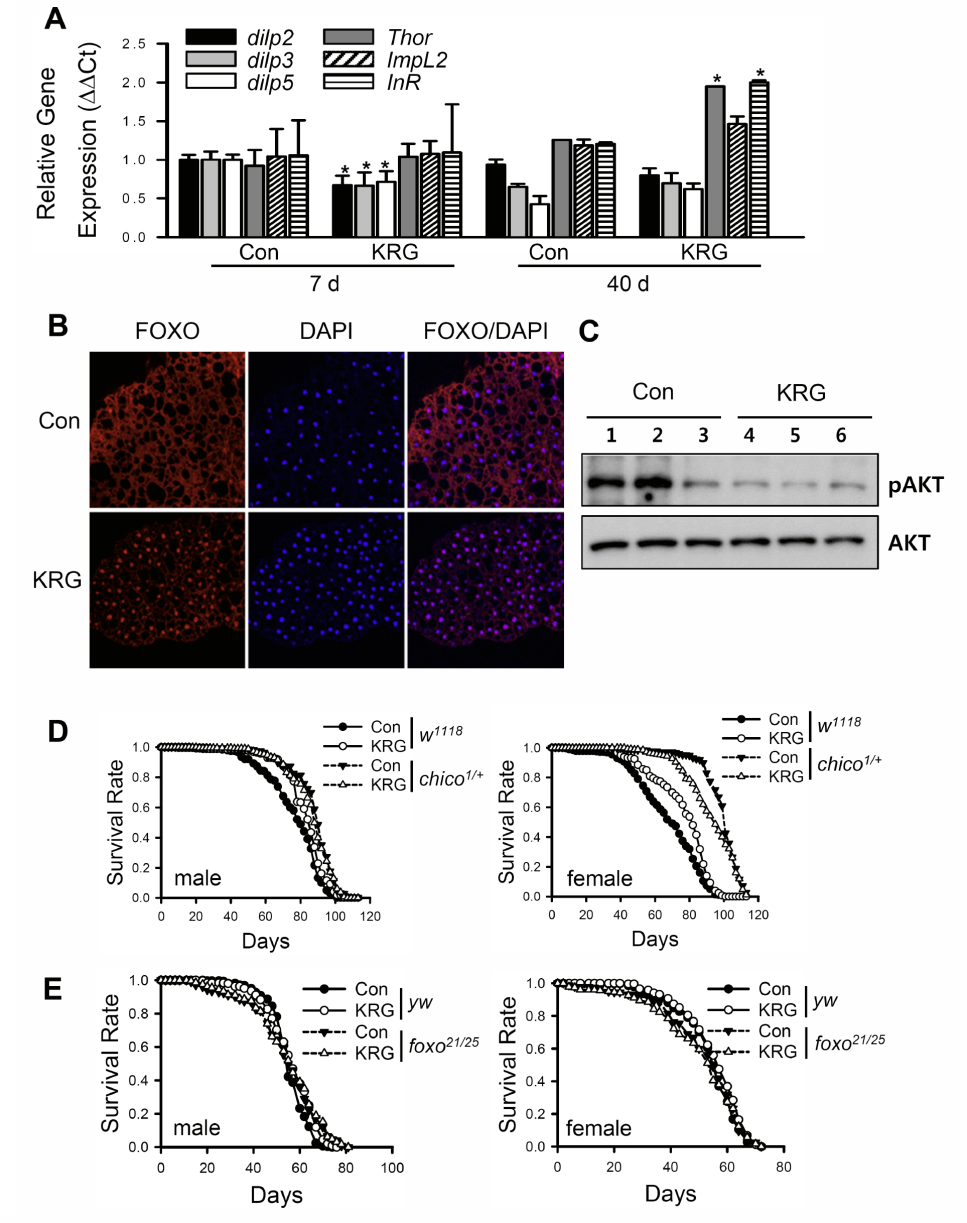
**Lifespan extension by KRG is mediated through the IIS pathway.** (**A**) mRNA levels of *dilps* and target genes of dFOXO were analyzed in fruit flies fed a KRG-containing diet or a control diet for 7 or 40 days. *p < 0.01. (**B**) Translocalization of dFOXO to the nucleus following supplementation of diet with KRG. The abdominal fat body was stained with anti-dFOXO (red) and DAPI (blue). Original magnification is 200×. (**C**) The level of phosphorylated Akt (pAkt)/Akt of flies fed a KRG-containing diet (lane 4, 5, 6) or a control diet (lane 1, 2, 3). (**D**) Survival of heterozygous mutant *chico* flies (*chico^1/+^*, dashed lines) and control flies (*w^1118^*, solid lines) fed a KRG-containing diet (lines with open dots) or a control diet (lines with closed dots). (**E**) Survival of transheterozygous mutant *dFOXO* flies (*foxo^21/25^*, dashed lines) and control flies (*yw*, solid lines) fed a KRG-containing diet (lines with open dots) or a control diet (lines with closed dots).

KRG-mediated health-improving effects such as anti-cancer, anti-diabetes, and anti-inflammation effects have been reported to be related to the insulin/IGF-1 signaling (IIS) pathway [33–35], and downregulation of IIS has been reported to increase lifespan in many model organisms [36, 37]. To verify whether KRG treatment can modulate the IIS pathway, the level of *Drosophila* insulin-like peptides (*dilps*) and the target genes of the dFOXO transcription factor, a component of IIS that is negatively regulated by the insulin receptor, were measured in flies fed KRG supplement. The mRNA levels of *dilps* were markedly decreased after KRG supplementation for 7 days, whereas the mRNA levels of FOXO target genes, such as *Thor* and *InR*, were significantly increased after 40 days of KRG supplementation (Figure 5A). *ImpL2* expression tended to be increased after 40 days of KRG supplementation, but the increase was not significant (Figure 5A, *t*-test *p* = 0.085). In addition, transcription factor dFOXO was translocated from cytoplasm to nucleus after KRG supplementation for 7 days (Figure 5B). Likewise, translocation of dFOXO to the nucleus was induced by DR, which is known to reduce IIS signaling (see Supplementary Figure 2). Furthermore, the level of phosphorylated Akt tended to decrease in the flies fed the KRG-supplemented diet, although the change had no statistical significance (Figure 5C and Supplementary Figure 3, *t*-test *p* = 0.106). These results indicate that KRG inactivates the IIS pathway.

To demonstrate whether the longevity effect of KRG is mediated by IIS, the lifespans of insulin receptor substrate mutant (*w^1118^;chico^1/+^*) or dFOXO mutant (*yw;foxo^21^/foxo^25^*) flies were determined after feeding a KRG supplement. Wild-type control flies (*w^1118^*) supplemented with KRG lived longer than non-treated control flies in both sexes (solid lines in Figure 5D and Supplementary Table 3). However, the lifespan of *w^1118^;chico^1/+^* males was unaffected by KRG supplementation (left panel of Figure 5D and Supplementary Table 3, log-rank test χ^2^ = 1.29, *p* = 0.26), whereas the lifespan of *chico^1/+^* females was decreased by KRG supplementation (right panel of Figure 5D and Supplementary Table 3, 6.26% decrease, log-rank test χ^2^ = 9.10, *p* < 0.01). In addition, a lifespan-extending effect of KRG was not detected in dFOXO mutant flies (Figure 5E and Supplementary Table 4, *yw* male, 2.78% increase, log-rank test χ^2^ = 13.45, *p* < 0.0001; *yw* female, 2.50% increase, log-rank test χ^2^ = 5.01, *p* < 0.05; *foxo^21/25^* male, log-rank test χ^2^ = 0.08, *p* = 0.77; *foxo^21/25^* female, log-rank test χ^2^ = 0.21, *p* = 0.65). These results indicate that the KRG-induced lifespan extension is mediated by a reduction in IIS signaling.

## DISCUSSION

*Panax ginseng*, one of the most popular CAMs worldwide, has been actively investigated for its nutraceutical benefits including anti-cancer, anti-diabetes, and anti-cardiovascular disease benefits; however, presence of a *P. ginseng* longevity benefit is unclear. In this study, the presence of a KRG longevity effect was verified by using a *Drosophila* model, and the lifespan benefit was shown to be associated with dSir2 and IIS.

The results of this study show that KRG supplementation at concentrations from 10 to 25 μg/mL can extend the lifespan of *D. melanogaster*. At doses greater than 25 μg/mL, KRG supplementation did not extend the lifespan; rather, it decreased the lifespan of female flies. These results can be explained by the presence of hormesis, a biphasic dose-response characterized by a low-dose beneficial effect and a high-dose toxic effect [38]. Many nutraceuticals and phytochemicals with prolongevity effects have been shown to reduce the lifespan of animals at high doses [23]. Zhang et al. [39] reported that panaxatriol saponins extracted from *P. notoginseng* improved movement behavior in Parkinson's disease model zebrafish at a low dose but produced neural toxicity at a high dose. Based on the hormetic nature of *P. ginseng*, the failure to detect lifespan extension in previous studies may be due to the use of high doses of ginseng extract; in fact, 0.25 mg/mL and 25 mg/mL extracts were supplied in one of the studies [19]. Interestingly, the concentrations of KRG tonic associated with the longevity benefit in a previous study were 1.2 μg/mL and 12 μg/mL [22], which are lower concentrations than those used in our results. One of the possible explanations for the observed differences is the different compositions of the nutraceutical ingredients used in this study and that used by Kim [22]. While our study used KRG extract alone, Kim [22] used a KRG tonic that contained several nutraceutical ingredients that are widely used for health improvement in Eastern countries.

In this paper, we assessed the effects of KRG on the resistance to thermal, starvation, and oxidative stresses. KRG supplementation did not affect the resistance to thermal stresses, such as heat-shock stress at 37°C and cold-shock stress at 0°C, but it did decrease the survival of flies under starvation stress conditions. Consistent with the decreased survival under starvation conditions, body weight (data not shown) and TAG level were decreased in young female flies by the administration of KRG. Similarly, several studies have shown that administration of *P. ginseng* can reduce body weight and lipid accumulation in *ob/ob* mice, fatty rats, obese zebrafishes, and humans [40–42]. Interestingly, our data showed that the effect of KRG on the TAG accumulation has a sex-specificity, *i.e*., KRG supplementation decreased the TAG level in females but increased it in males. This differential TAG level response to KRG supplementation may be caused by differences in lipid metabolism between male and female flies. Compared to males, females have a higher lipid content, particularly in mature oocytes, one of the major lipid storage areas [43], and that content is sex-specifically controlled by several hormones such as insulin, juvenile hormone, and ecdysone. In addition, gene expression was reported to be changed by a high-fat diet treatment, with sex-dependency, in *Drosophila* [44].

In addition, our data showed that KRG supplementation can increase survival under oxidative stress. For the measurement of oxidative stress resistance, we undertook the oral administration of paraquat, a chemical that catalyzes the production of superoxide anions. Although this method has limitations because the induction level of oxidative stress is dependent upon the feeding behavior level, it has been widely used for the induction of oxidative stress in many model organisms including *Drosophila* [23, 45, 46] and we observed that KRG supplementation did not affect the feeding behavior of flies, indicating that the resistance of KRG-supplemented flies to paraquat feeding was not the result of a feeding artifact. Our data also showed that KRG supplementation increases the expression and the activity of antioxidant enzymes, similar to results in reports showing increases in antioxidant levels associated with KRG supplementation in mice, rats, and humans [30, 47–49]. Many phytochemicals including resveratrol, curcumin, epigallocatechin-3-*O*-gallate, and epicatechin are known to activate redox-sensitive nuclear factor-E2-related factor 2 (Nrf2) signaling [50, 51]. Similarly, ginseng and ginsenosides activate Nrf2 signaling, and its activation has a role in the induction of antioxidant enzymes by ginseng and its components [52, 53]. Moreover, the increases in antioxidant enzymes such as SOD, GSH, and GST induced by ginseng or red ginseng-specific ginsenoside Rh2 have been reported to be mediated by the expression of Nrf2 in rats with cyclophosphamide-induced liver damage [52] and in mice with lipopolysaccharide-induced lung damage [53], respectively.

DR, the reduction of caloric intake without malnutrition, has been widely reported to improve health and to extend lifespan in many organisms including yeast, nematodes, fruit flies, rodents, and primates [31]. The anti-aging effects of DR have been reported to be associated with the activation of the NAD+ dependent deacetylase Sirt1 [54]. In our results, KRG supplementation extended the lifespan of fruit flies that ingested a full diet but did not extend the lifespan of flies that ingested a restricted diet. In addition, KRG supplementation suppressed the age-related decrease of *dSir2* expression, and the lifespan-extending effect of KRG was completely repressed by the mutation of *dSir2*. Taken together, these results indicate that the KRG-induced life extension is mediated by dSir2, in a manner similar to the mechanism associated with DR; however, we cannot exclude the possibility of a non-specific effect caused by the general detrimental consequences of the short-lived *dSir2* mutant. Consistent with our results, a recent report showed that Sirt1 protein expression could be induced by treatment of A549 cells with a water extract of KRG at 500 μg/mL [55]; moreover, feeding with KRG at 250 and 500 mg/kg for 4 weeks increased the expression of Sirt1 in alcoholic liver disease model mice [56]. In addition, panaxatriol saponin from *P. notoginseng* was reported to have a neuroprotective effect through AMPK/SIRT1/FOXO3 signaling pathway in zebrafish [39]. However, the present study is the first to report a role of Sirtuin in the prolongevity effect of KRG.

In our study, we showed that KRG extends the lifespan of flies via *dSir2* expression in a manner similar to that of DR. However, not all of the changes induced by DR were induced with KRG supplementation. For example, the steady-state TAG level of animals experiencing DR is reported to be increased [57]; in contrast, KRG decreased the level of TAG in female flies in our study. However, the overexpression of *dSir2* in the larval fat body has shown to be related to a depletion of fat storage [58], which is in line with our data showing increased *dSir2* expression and decreased fat storage in females. These results suggest that KRG partially mimics the Sirtuin-related DR effect.

A role of IIS in the regulation of lifespan has been reported in various model organisms [36, 37]. Ginseng has been actively investigated for its role in regulating the IIS pathway, and the nutraceutical effects of ginseng on several aging-related diseases are reported to be mediated by its modulation of IIS. For example, KRG has improved insulin sensitivity in rats fed a high-fat diet [33], and total saponin from KRG was reported to reduce the thrombin-induced phosphorylation of PI3K and Akt in human platelets [35]. In addition, ginsenoside Rg3 has reduced tumor volume and weight in xenograft model mice, results that were considered to be related to inhibition of the PI3K/Akt signaling pathway [59]. Moreover, the protopanaxadiol ginsenoside Rb1 suppressed LPS-induced Akt phosphorylation in the human fetal microglial cell line [60]. Consistent with the results in these previous reports, our results show that the longevity benefit of KRG is related to the IIS pathway. In this study, KRG supplementation reduced the expression of insulin-like peptides and increased the activity of dFOXO. In addition, the life-extending effect of KRG was diminished by mutation of *chico* or *dfoxo*. These results indicate that the longevity benefit of KRG is mediated by the reduction of IIS signaling. Unlike the results obtained from *Drosophila*, lifespan extension by ginseng was unaffected in a *daf-16* (encoding the FOXO) mutant of *C. elegans* [16]. The evolutionarily conserved mechanisms associated with ginseng-induced lifespan extension need to be elucidated through future studies.

IIS and DR are reported to be associated with distinct cellular mechanisms involved in extending lifespans in *C. elegans* and *D. melanogaster* [61, 62], but several studies have shown that some physiological effects of DR, such as intestinal vesicle trafficking and growth impairment, are suppressed in *daf-2* (encoding the insulin-like growth factor 1) mutation, and that *daf-2* mutation modulates the prolongevity effect of DR in *C. elegans* [63], suggesting the presence of partially overlapping lifespan extension mechanisms in IIS and DR. Our results have shown that the prolongevity effect of KRG is mediated by both DR and IIS in *D. melanogaster*. It will be intriguing future work to unveil the crosstalk between Sir2/DR and the IIS pathway within the prolongevity function of KRG and to examine the evolutionarily conserved mechanism of KRG lifespan extension in rodent models.

*Panax ginseng* has a variety of bioactive components such as ginsenosides, polysaccharides, sugar, and amino acids, and these compositions vary depending on the harvesting season, extraction methods, and cultivating environment. The ginsenosides Rb1, Rg1, Rc, Rb2, and Re were abundant in the red ginseng extract used in this study and have been reported to prevent cellular senescence induced by H_2_O_2_ exposure in astrocyte [64]. In addition, ginsenoside Rc was reported to be the major component of *Panax ginseng* that prolongs the lifespan of *C. elegans* [17], and ginsenoside Rg3 can activate Sirtuin deacetylase activity in rat [65]. Further studies to examine the role of specific ginsenosides on lifespan extension will help to elucidate the major components of *Panax ginseng* that are active in extending lifespan and to develop novel DR mimetic.

## MATERIALS AND METHODS

### Drosophila stocks and husbandry

All experiments were performed with CS_10_ (*w^1118^* outcrossed 10 times to Canton-S) *D.* *melanogaster* except for mutant experiments that used mutant fruit flies. The *w^1118^;Sir2^2A-7-11^*, *chico^1^/CyO*, and the background *w^1118^* flies were obtained from the Bloomington Drosophila Stock Center at Indiana University, while the *yw;;foxo^25^/TM6B Tb* and *yw;;foxo^21^/TM6B* were provided by K Yu (KRIBB, Korea). The *w^1118^;chico^1/+^*, obtained from a cross of the *chico^1^/CyO* males to the *w^1118^* females, was used for the *chico* mutant experiment. All flies were cultured and reared at 25°C and 65% humidity on 12:12 hour light:dark cycles. To avoid larval crowding, approximately 150 eggs were laid on 250 cm^3^ fly bottles containing 25-30 mL of medium and were developed until eclosion to adult.

Standard cornmeal-sugar-yeast with agar (CSY) medium including 5.2% cornmeal (Hansol Tech, Korea), 11% sugar (Hansol Tech), 2.5% Saf-instant yeast (Lesaffre, France), 0.5% propionic acid (Junsei Chemical Co. Ltd., Japan), 0.04% methyl-4-hydroxybenzoate (Yakuri Pure Chemicals Co. Ltd., Japan), and 0.8% agar (Milyang Agar Co. Ltd., Korea) was used to rear fly larvae, and when flies were eclosed as adults, standard sugar-yeast (SY) medium including 10% sugar, 10% Saf-instant yeast, 0.5% propionic acid, 0.04% methyl-4-hydroxybenzoate, and 0.8% agar was provided [23]. In the dietary-restriction experiment, yeast extracts (Duchefa Biochemie, Netherlands) were used for SY medium at concentrations of 1%, 4%, or 8%, as required. We previously reported that 1% and 4% yeast extracts in SY medium were sufficient to produce a longevity benefit through DR compared to 8% yeast extract in fruit flies [66].

### Supplementation of KRG

KRG powder was provided by the Korean Tobacco & Ginseng Corporation (Daejeon, Korea). To produce the KRG powder, roots from 6-year-old *P. ginseng* Mayer were processed by steaming and drying. The ginsenoside content of the supplied KRG was Rg1: 3.21 mg/g, Rb1: 6.44 mg/g, Rg3s: 0.18 mg/g, Re: 2.08 mg/g, Rc: 2.68 mg/g, Rb2: 2.25 mg/g, Rd: 0.5 mg/g, Rf: 0.89 mg/g, Rh1: 0.21 mg/g and Rg2s: 0.29 mg/g (information provided by supplier). The KRG powder was dissolved in distilled water and added to SY food during food preparation at final concentrations of 10, 25, or 50 μg/mL. The KRG concentrations were selected based on our preliminary studies and a previous report [22]. The KRG-supplemented food (4 mL) at indicated concentrations was delivered to individual *Drosophila* polypropylene vials (25 mm diameter, 95 mm height, Hansol Tech). After 24 hours, 2- to 3-day-old adult flies were transferred to the vials containing KRG-supplemented food for the indicated period in each experiment.

### Lifespan assays

For the conventional lifespan assay (Figure 1), newly eclosed 100 male and 100 fertilized female adult fruit flies were transferred to a 500 cm^2^ demography cage using CO_2_ anesthesia. For the lifespan assay under DR condition (Figure 3), 2- to 3-day-old 100 males or 100 fertilized females were separately housed in a demography cage. Three replicate cages were set up for each group. Vials containing fresh SY food with/without KRG were affixed to cages and changed every 2 days, at which time dead flies were removed and recorded. The Kaplan-Meier survival estimator was used to estimate the survival function from the lifetime data, and log-rank tests were carried out to determine the statistical significance of differences in mean lifespan. The JMP statistical package (SAS, USA) and SPSS (SPSS Inc., USA) was used for the analyses. This lifespan experiment was independently performed twice, and we observed that the results were reproducible.

### Measurement of fecundity

Male and female fruit flies were collected separately every 3 h after initiation of the first eclosion. Female virginity was confirmed via the absence of progeny in the food after 24 h. Vials were set up on day 2 with a population density of 2 males and 1 virgin female. Every subsequent 24 h, the flies were transferred to new vials containing fresh SY food with/without KRG, and the daily number of eggs laid by each female was counted for 10 days. Twenty replicate vials were tested per treatment. Statistical probability was determined by using the *t*-test. The fecundity experiment was independently performed twice.

### Measurement of feeding amount

The feeding amount assay was performed as previously reported but with minor modifications [67]. After feeding the KRG-supplemented diet for 7 days, 10 single-sex flies were transferred to a new vial containing the same diet with blue-dye number 1 (0.05% wt/vol) added. After feeding for 15 min, 1 h, or 4 h, anesthetized flies were washed with phosphate-buffered saline (PBS) and homogenized in 0.2 mL distilled water. After centrifugation for 5 min at 13,000 r/min, the absorbance of the supernatants was measured at 595 nm with a spectrophotometer (Sunrise, Tecan, Austria). Ten replicate vials were tested per treatment. Statistical probability was determined by using the *t*-test. This experiment was independently performed at least three times.

### Measurement of climbing ability

Adult fruit flies were fed SY food with/without KRG for 7 or 21 days prior to performing a vertical climbing assay. At the time point indicated, 10 single-sex flies were loaded into the vertical climbing assay apparatus, which was then tapped on a tabletop three times in rapid succession to initiate negative geotaxis responses in the flies. The positions of the flies in the tubes were captured by digital images taken 4 sec after initiating the geotactic behavior, and the number of flies at 4 cm above the apparatus bottom was determined. The flies were assessed in consecutive trials separated by 1 min of rest. Twenty replicates were used in four experiments per treatment. Statistical probability was determined by using the *t*-test.

### Stress resistance test

### Thermal stress

After feeding with the KRG supplement for 7 days, 10 single-sex fruit flies were transferred to a new vial. For the heat-shock stress test, flies were exposed to heat (37°C) by sinking the vial in a water bath. Numbers of dead flies were recorded every 10 min until all flies were dead. Fifteen replicates were established. For the cold-shock stress test, flies were exposed to cold (0°C) for 8 h, returned to room temperature, and the recovery duration (minutes) until their awakening was recorded. Ten replicates were established.

### Starvation stress

After feeding with the KRG supplement for 7 days, 10 single-sex fruit flies were transferred to vials containing 0.8% agar every 6 h. Dead flies were counted after each transfer. Fifteen replicates were established.

### Oxidative stress

After feeding with the KRG supplement for 7 days, 10 single-sex fruit flies were kept in vials containing 5% sucrose (Sigma-Aldrich, USA) and 15 mM paraquat (methyl viologen dichloride hydrate, CAS number 75365-73-0, 98% purity, Sigma-Aldrich). Every 4 h, the flies were transferred to fresh vials containing the paraquat/sucrose medium, and the dead flies were counted after each transfer. Fifteen replicates were established.

### Measurement of triacylglycerol

The level of triacylglycerol (TAG) was measured as previously described [68]. Newly eclosed flies were pretreated with 25 μg/mL KRG for 10 days. Heat-treated homogenized samples from 20 flies were used for the measurement of TAG and free glycerol levels by using TAG reagent (T2449, Sigma-Aldrich) and free glycerol reagent (F6428, Sigma-Aldrich). Since body weight can be easily affected by water content, TAG level was normalized to the protein content.

### Real-time quantitative PCR

After feeding fruit flies with 25 μg/mL KRG for 7 or 40 days, total RNA was extracted from 15 male flies by using RNAiso (Takara Bio, Japan). Total RNA (2 μg) was reverse transcribed by using M-MLV reverse transcriptase (Promega, USA). Quantitative PCR was performed by using the Prism 7500 Sequence Detection System (Applied Biosystem, USA) and TOPreal^TM^ qPCR 2× PreMix (Enzynomics, Korea) following the manufacturer's instructions. At least three replicates were established for each group, and all experiments were repeated at least three times. Data are presented as mean ± standard error of mean values. *Ribosomal protein 49* (*rp49*) was used as the internal control. The primer oligonucleotide sequences are available in Supplementary Information.

### Measurement of antioxidant enzymes activity

Newly eclosed male flies were pretreated with KRG 25 μg/mL for 7 days. Thirteen to thirty flies with three replicates were collected with CO_2_ anaesthetization. Activities of superoxide dismutase (SOD) and catalase (Cat) were measured using the SOD Assay Kit-WST (19160, Sigma-Aldrich) and Catalase Activity Colorimetric Assay Kit (BioVision, USA), respectively, as described by manufacturers. Statistical probability was measured using *t*-test.

### Immunohistochemistry

Adult female flies supplemented with the KRG supplement for 7 days were dissected and the adult fat body attached to the abdominal cuticles was isolated in 4% formaldehyde (Sigma-Aldrich). After fixation with 4% formaldehyde for 1 h and washing with PBST (PBS + 0.1% Triton X-100), samples were incubated with primary antibodies in PBST-2% bovine serum albumin (BSA) at 4°C overnight. Samples were then washed with PBST, incubated with secondary antibodies for 60 min at 25°C, washed with PBST, and mounted with Vectashield mountant (Vector Labs, USA). Anti-dFOXO antibody (a gift from O. Puig) and Cy^TM^3-conjugated anti-rabbit IgG secondary antibody (Jackson Immuno Research Laboratories, USA) were diluted to 1:500 and 1:300, respectively, in PBST-2%BSA solution. DAPI was used to counterstain the nuclei. The resulting images were assessed by using a confocal laser scanning microscope (LSM510 META, Carl Zeiss, Germany).

### Western blot analysis and quantification

After feeding fruit flies with 25 μg/mL KRG for 7 days, total proteins from male flies were lysed by PRO-PREP protein extraction buffer (iNtRON biotechnology, South Korea) and centrifuged 10 min at 13,000 r/min. Supernatants of samples were quantified using Bradford assay and equal amount of protein was loaded. Western Blot was performed with rabbit *Drosophila* phospho-Ser505 Akt (1:2000, Cell Signaling, USA), rabbit *Drosophila* Akt (1:2000, Cell Signaling) antibodies and anti-rabbit IgG (1:5000, Santa Cruz, USA) secondary antibody was used. Phospho-Ser505 Akt/Akt ratio was quantified using ImageJ software.

## Supplementary Material

Supplementary Materials

Supplementary Figures

Supplementary Tables
