# Supplementary material for: Extension of Drosophila lifespan by Korean red ginseng through a mechanism dependent on dSir2 and insulin/IGF-1 signaling
Source: Aging (Albany NY). 2019 Oct 31;11(21):9369–87. doi: 10.18632/aging.102387 (PMC6874434; doi:10.18632/aging.102387)
Supplement: Supplementary Materials [file aging-11-102387-s001.pdf]

SUPPLEMENTARY METHODS

Primers for Quantitative PCR

The primer oligonucleotide sequences for the *Sod1* [1], *Sod2* [2], *Cat* [1], *dSir2* [3], *Thor* [4], *InR* [5], *ImpL* [6], *dilp2* [7], *dilp3* [7], *dilp5* [7], and *rp49* [8] analyses are as follows:
Sod1: forward, 5′-GTT CGG TGA CAA CAC CAA TG-3′, reverse, 5′-GGA GTC GGT GAT GTT GAC CT-3′; *Sod2*: forward, 5′-TGT TCG TGG CCC GTA AAA T-3′; reverse, 5′-GTC TGG TGC TTC TGG TG-3′; *Cat*: forward, 5′-TAC GAG CAG GCC AAG TT-3′, reverse, 5′-ACC TTG TAC GGG CAG TTC AC-3′; *dSir2*: forward, 5′-CGC ACA CCA ACA ACT ATT CG-3′, reverse, 5′- CGG TAG CAA TGG TGA CAA TG-3′; *Thor*: forward, 5′-GAA GGT TGT CAT CTC GGA TCC-3′, reverse, 5′-ATG AAA GCC CGC TCG TAG A-3′; *InR*: forward, 5′-AAC AGT GGC GGA TTC GGT T-3′, reverse, 5′-ACT CGG AGC ATT GGA GGC AT-3′; *ImpL*: forward, 5′-GCC GAT ACC TTC GTG TAT CC-3′, reverse, 5′-TTT CCG TCG TCA ATC CAA TAG-3′; *dilp2*: forward, 5′-ACG AGG TGC TGA GTA TGG TGT CGC-3′, reverse, 5′-CAC TTC GCA GCG GTT CCG ATA TCG-3′; *dilp3*: forward, 5′- CAG GCC ACC ATG AAG TTG TGC-3′, reverse, 5′-CTT TCC AGC AGG GAA CGG TCT TCG-3′; *dilp5*: forward, 5′-TGT TCG CCA AAC GAG GCA CCT TGG-3′, reverse, 5′-CAC GAT TTG CGG CAA CAG GAG TCG-3′; *rp49*: forward, 5′-TCC TAC CAG CTT CAA GAT GAC-3′, reverse, 5′-CAC GTT GTG CAC CAG GAA CT-3′.

Measurement of dehydration

The level of water content was measured as described (9). Newly eclosed flies were pretreated with 25 μg/mL KRG for 10 days. Ten flies per sample were weighted, dried for 48 h at 70°C, and then weighed again. The difference in weights divided by the initial weight was taken to be the water content. Fifteen replicates were used per treatment. Statistical probability was determined by using the *t*-test.

SUPPLEMENTARY REFERENCES

1. Tsuda M, Ootaka R, Ohkura C, Kishita Y, Seong KH, Matsuo T, Aigaki T. Loss of Trx-2 enhances oxidative stress-dependent phenotypes in Drosophila. FEBS Lett. 2010; 584:3398–401. https://doi.org/10.1016/j.febslet.2010.06.034 PMID:20600005

2. Shen LR, Xiao F, Yuan P, Chen Y, Gao QK, Parnell LD, Meydani M, Ordovas JM, Li D, Lai CQ. Curcumin-supplemented diets increase superoxide dismutase activity and mean lifespan in Drosophila. Age (Dordr). 2013; 35:1133–42. https://doi.org/10.1007/s11357-012-9438-2 PMID:22653297

3. Lee SH, An HS, Jung YW, Lee EJ, Lee HY, Choi ES, An SW, Son H, Lee SJ, Kim JB, Min KJ. Korean mistletoe (Viscum album coloratum) extract extends the lifespan of nematodes and fruit flies. Biogerontology. 2014; 15:153–64. https://doi.org/10.1007/s10522-013-9487-7 PMID:24337961

4. Flatt T, Min KJ, D'Alterio C, Villa-Cuesta E, Cumbers J, Lehmann R, Jones DL, Tatar M. Drosophila germ-line modulation of insulin signaling and lifespan. Proc Natl Acad Sci U S A. 2008; 105:6368–73. https://doi.org/10.1073/pnas.0709128105 PMID:18434551

5. Fuss B, Becker T, Zinke I, Hoch M. The cytohesin Steppke is essential for insulin signalling in Drosophila. Nature. 2006; 444:945–8. https://doi.org/10.1038/nature05412 PMID:17167488

6. Rera M, Clark RI, Walker DW. Intestinal barrier dysfunction links metabolic and inflammatory markers of aging to death in Drosophila. Proc Natl Acad Sci U S A. 2012; 109:21528–33. https://doi.org/10.1073/pnas.1215849110 PMID:23236133

7. Okamoto N, Yamanaka N, Yagi Y, Nishida Y, Kataoka H, O'Connor MB, Mizoguchi A. A fat body-derived IGF-like peptide regulates postfeeding growth in Drosophila. Dev Cell. 2009; 17:885–91. https://doi.org/10.1016/j.devcel.2009.10.008 PMID:20059957

8. Biteau B, Hochmuth CE, Jasper H. JNK activity in somatic stem cells causes loss of tissue homeostasis in the aging Drosophila gut. Cell Stem Cell. 2008; 3:442–55. https://doi.org/10.1016/j.stem.2008.07.024 PMID:18940735

9. Schriner SE, Lee K, Truong S, Salvadora KT, Maler S, Nam A, Lee T, Jafari M. Extension of Drosophila lifespan by Rhodiola rosea through a mechanism independent from dietary restriction. PLoS One. 2013; 8:e63886. https://doi.org/10.1371/journal.pone.0063886 PMID:23704949
