# Supplementary material for: Extension of Drosophila lifespan by Korean red ginseng through a mechanism dependent on dSir2 and insulin/IGF-1 signaling
Source: Aging (Albany NY). 2019 Oct 31;11(21):9369–87. doi: 10.18632/aging.102387 (PMC6874434; doi:10.18632/aging.102387)
Supplement: Supplementary Figures [file aging-11-102387-s002.pdf]

SUPPLEMENTARY FIGURES

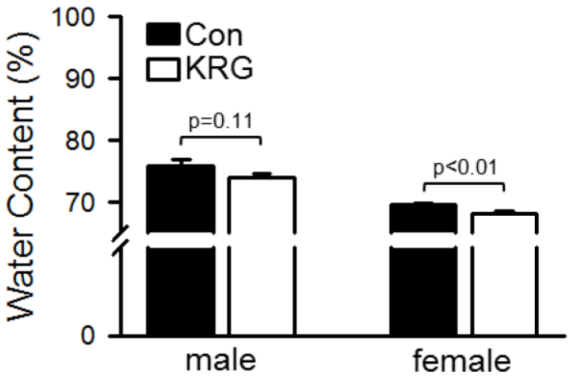

Supplementary Figure 1. The water content of KRG treated flies. The level of water content of flies fed a KRG-containing diet (white bars) or a control diet (black bars). Supplementation of KRG extract decreased the water content in female flies (*t*-test).

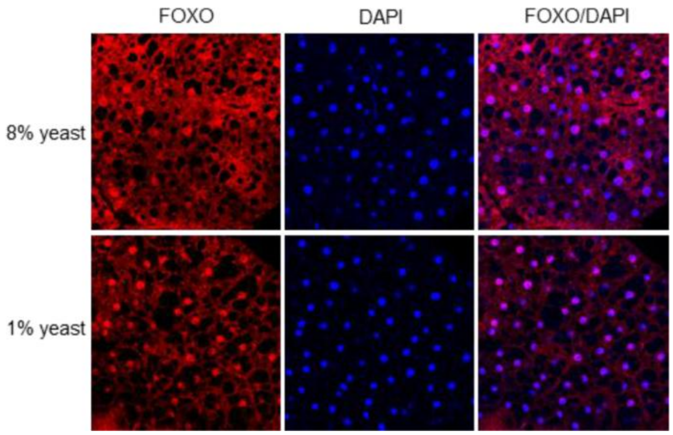

Supplementary Figure 2. Translocalization of dFOXO into nucleus by DR. Abdominal fat body of 7-day-old flies fed full diet (8% yeast) or restricted diet (1% yeast) was stained with anti-dFOXO (red) and DAPI (blue). Original magnification is 200×.

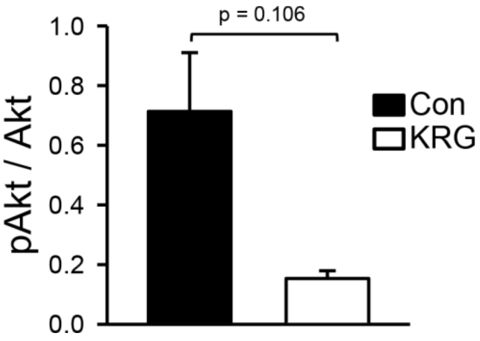

Supplementary Figure 3. pAkt/Akt level of flies fed KRG. The level of phosphorylated Akt (pAkt) normalized with total Akt in the flies fed a KRG-containing diet (white bar) or a control diet (black bar). Supplementation of KRG extract decreased the pAkt/Akt level but do not have a statistical significance (*t*-test, $p = 0.106$).
