# Supplementary material for: Extension of Drosophila lifespan by Korean red ginseng through a mechanism dependent on dSir2 and insulin/IGF-1 signaling
Source: Aging (Albany NY). 2019 Oct 31;11(21):9369–87. doi: 10.18632/aging.102387 (PMC6874434; doi:10.18632/aging.102387)
Supplement: Supplementary Tables [file aging-11-102387-s003.pdf]

SUPPLEMENTARY TABLES

Supplementary Table 1. Lifespan of *Drosophila melanogaster* fed a KRG-supplemented diet.

Sex	KRG (μg/mL)	Mean lifespan (days)	Median lifespan (days)	*p*-value	χ^2	N
Male	0	29.89 ± 0.70	31			254
	10	33.86 ± 0.57	35	0.031*	4.641	260
	25	34.18 ± 0.56	35	0.004*	8.159	267
	50	31.46 ± 0.65	33	0.365	0.822	270
Female	0	22.01 ± 0.42	22			303
	10	21.51 ± 0.36	22	0.058	3.589	292
	25	23.81 ± 0.40	24	0.018*	5.633	284
	50	19.53 ± 0.36	19	<0.0001*	24.600	291

*Asterisks indicate significant differences compared to control (Log-rank test). Mean lifespan is presented as the average lifespan ± standard error. N indicates fly numbers analyzed.

Supplementary Table 2. Lifespan of *D. melanogaster* fed various yeast extract diets with or without KRG supplementation.

Sex	Yeast conc. (%)	KRG (μg/mL)	Mean lifespan (days)	Median lifespan (days)	*p*-value	χ^2	N
Male	1	0	58.32 ± 0.91	61	0.012*	6.342	269
		25	57.30 ± 0.80	59			259
	4	0	52.26 ± 0.97	53	0.331	0.945	266
		25	51.93 ± 0.88	55			266
	8	0	45.20 ± 0.96	47	0.032*	4.579	273
		25	48.12 ± 0.89	49			270
Female	1	0	46.99 ± 0.98	49	0.466	0.531	283
		25	47.58 ± 0.85	49			278
	4	0	38.20 ± 0.87	41	0.013*	6.164	271
		25	41.85 ± 0.82	43			274
	8	0	27.77 ± 0.72	27	0.012*	6.275	296
		25	29.55 ± 0.81	31			273

*Asterisks indicate significant differences compared to control (Log-rank test). Mean lifespan is presented as the average lifespan ± standard error. N indicates fly numbers analyzed.

Supplementary Table 3. Effect of KRG diet supplement on the lifespan of *dSir2* and *chico* mutant fruit flies.

Sex	Strain	KRG (μg/mL)	Mean lifespan (days)	Median lifespan (days)	*p*-value	χ^2	N
Male	*w*^{1118}	0	76.47 ± 1.01	80	<0.0001*	18.417	253
		25	83.11 ± 0.80	86			257
	dSir2$^{2A-7-11}$	0	47.90 ± 0.75	48	0.280	1.169	268
		25	45.96 ± 0.83	46			272
	chico$^{1/+}$	0	88.06 ± 0.83	90	0.257	1.287	236
		25	86.67 ± 0.80	89			262
Female	*w*^{1118}	0	67.62 ± 1.11	70	<0.0001*	28.885	291
		25	76.55 ± 0.98	82			278
	dSir2$^{2A-7-11}$	0	48.89 ± 0.93	53	0.011*	6.387	266
		25	45.82 ± 0.91	48			281
	chico$^{1/+}$	0	99.15 ± 0.77	101	0.003*	9.096	248
		25	92.94 ± 0.97	95			249

*Asterisks indicate significant differences compared to control (Log-rank test). Mean lifespan is presented as the average lifespan ± standard error. N indicates fly numbers analyzed.

Supplementary Table 4. Effect of KRG diet supplement on the lifespan of *dFOXO* mutant fruit flies.

Sex	Strain	KRG (μg/mL)	Mean lifespan (days)	Median lifespan (days)	*p*-value	χ^2	N
Male	*yw*	0	55.07 ± 0.56	55	<0.0001*	13.453	287
		25	56.60 ± 0.69	57			249
	foxo$^{21/+}$	0	59.14 ± 0.69	60	0.003*	8.784	247
		25	61.12 ± 0.77	64			262
	foxo$^{25/+}$	0	67.04 ± 0.60	70	0.245	1.351	286
		25	64.94 ± 0.75	67			251
	foxo$^{21/25}$	0	54.68 ± 0.92	57	0.772	0.084	280
		25	55.07 ± 0.88	57			294
Female	*yw*	0	52.30 ± 0.77	55	0.025*	5.012	295
		25	53.61 ± 0.85	57			251
	foxo$^{21/+}$	0	54.28 ± 0.67	57	0.092	2.836	276
		25	54.93 ± 0.66	55			270
	foxo$^{25/+}$	0	62.57 ± 0.72	64	0.221	1.499	273
		25	64.18 ± 0.67	64			250
	foxo$^{21/25}$	0	52.99 ± 0.84	55	0.646	0.210	227
		25	52.26 ± 0.86	55			218

*Asterisks indicate significant differences compared to control. Mean lifespan is presented as the average lifespan ± standard error. N indicates fly numbers analyzed.
